# Supplementary material for: Alkali-deficiency driven charged out-of-phase boundaries for giant electromechanical response
Source: Nat Commun. 2021 May 14;12:2841. doi: 10.1038/s41467-021-23107-x (PMC8121868; doi:10.1038/s41467-021-23107-x)
Supplement: Supplementary file 1 — Supplementary Information [file 41467_2021_23107_MOESM1_ESM.pdf]

Supplementary Information for

Alkali-deficiency driven charged out-of-phase boundaries for giant electromechanical response

Haijun Wu,[1,2,#,*] Shoucong Ning,[2,#] Moaz Waqar,[2,#] Huajun Liu,[3] Yang Zhang,[4] Honghui Wu,[5,*] Ning Li,[2] Yuan Wu,[5] Kui Yao,[3] Turab Lookman,[6] Xiangdong Ding,[1] Jun Sun,[1] John Wang,[2,*] Stephen J. Pennycook[2,*]

[1] State Key Laboratory for Mechanical Behavior of Materials, Xi'an Jiaotong University, Xi'an 710049, China.

[2] Department of Materials Science and Engineering, National University of Singapore, 9 Engineering Drive 1, 117575, Singapore.

[3] Institute of Materials Research and Engineering, A*STAR (Agency for Science, Technology and Research), 2 Fusionopolis Way, 138634, Singapore.

[4] Instrumental Analysis Center of Xi'an Jiaotong University, Xi'an Jiaotong University, Xi'an 710049, China.

[5] State Key Laboratory for Advanced Metals and Materials, University of Science and Technology Beijing, Beijing, China.

[6] 818 Bishops Lodge Road, Santa Fe, New Mexico, 87501, USA.

[*] Corresponding author.

Email address: wuhaijunnavy@xjtu.edu.cn (H. J. Wu); wuhonghui@ustb.edu.cn (H. H. Wu); msewangj@nus.edu.sg (J. Wang); stevepennycook@gmail.com (S. J. Pennycook);

[#] Equal contribution

Supplementary methods

Thin Film Fabrication

Different thicknesses of $NaNbO_3$ (NNO) films were deposited on (001) oriented 0.5% Nb-doped $SrTiO_3$ (Nb: STO) substrates using Radio Frequency Magnetron Sputtering. The deposition was carried out at 680 °C for 10 min, 2 hours, and 4 hours at 650 °C with Ar: O_2 =

2 : 1, power of 100 W (3-inch target), and a total pressure of 0.03 mbar. After deposition, thin films were cooled under the same gas flow as the deposition condition inside the chamber.

STEM and EELS Measurements

Plan view samples were prepared by polishing the samples followed by argon ion milling from the substrate side using an ion miller (Fischione M1051 TEM Mill). Cross-section samples were prepared using a focused ion beam (FIB) milling (FEI Versa 3D microscope). The samples were thinned using successive milling by 30 kV, 8 kV and 5 kV ion beams where a 2 kV beam was used for final cleaning. STEM imaging measurements were done using a JEOL ARM200F atomic resolution electron microscope equipped with a cold field emission gun, an ASCOR 5th order aberration corrector under an acceleration voltage of 200 kV. HAADF images were acquired using inner and outer collection semi-angles of 68 and 280 mrad respectively with a convergence semi-angle of 22 mrad. ABF images were acquired using a collection semi-angle of 17 mrad. STEM HAADF and ABF images were average background subtraction filtered for improved contrast. Atomic displacement was measured using MacTempas software and a prewritten script on MATLAB. Atomic models were made using Vesta software. STEM-DPC was performed on Thermo Scientific Spectra 300.

<u>Density functional theory (DFT) calculations</u>

The DFT calculations are performed using the projector augmented-wave (PAW) method as implemented in the Vienna ab initio simulation package (VASP) code. The exchange-correlation interaction is treated in the generalized gradient approximation (GGA) of Perdew-Burke-Ernzerhof (PBE) functionals. The Kohn-Sham orbitals are expanded in plane waves with a kinetic energy cutoff of 500 eV. A supercell consisting of 662 atoms with NNO film on $SrTiO_3$ film is used to conduct formation energy calculations, and the k-point sampling and Brillouin zone integration are done with Gamma grid points. The convergence criterion for electronic self-consistent calculations is 10^{-5} eV. Both atomic positions and lattice parameters are allowed to change during the structural relaxation until Hellmann–Feynman forces of all atoms are lower than 0.05 eV/Å.

The stability of an anti-site atom is evaluated via its formation energy, which is defined by

$$\Delta H_{d,q} = E_{d,q} - E_{pure} - \sum_i n_i(E_i + \mu_i) + q(E_V + E_F) \quad \text{(S1)}$$

where $E_{d,q}$, and E_{pure} are the total energy of the defect system and the host perfect supercell, respectively. n_i and E_i are the numbers and total energy of the component atom i added to (positive n_i), or taken from (negative n_i) the bulk perfect supercell to create the defect. μ_i are the corresponding chemical potentials of the constituent species, depending on experimental conditions. For a maximally rich growth environment of element i, $\mu_i = 0$. q is the number of electrons transferred from the supercell to the reservoirs in forming the defect cell. E_F is the Fermi level with respect to the energy of the valence band maximum (VBM, E_V) of the ideal supercell.

Phase-field simulations

In phase-field modelling of ferroelectric materials, the temporal evolution of the ferroelectric polarization P_i (i=1, 2, 3) is described by solving the time-dependent Ginzburg-Landau equation,

$$\frac{\partial P_i(\mathbf{r},t)}{\partial t} = -L\frac{\delta F_{\text{total}}}{\delta P_i(\mathbf{r},t)} \quad \text{(S2)}$$

where L is the kinetic coefficient, t is the time, $\delta F_{\textbf{total}} / \delta P_i(\mathbf{r},t)$ is the thermodynamic driving force for the spatial and temporal evolution of $\partial P_i(\mathbf{r},t)$, $\mathbf{r}$ =(x_1, x_2, x_3) denotes the spatial vector, and F_{total} is the total free energy over the simulated system,

$$F_{\text{total}} = \iiint_V \left(f_{bulk} + f_{\text{electrostatic}} + f_{gradient} + f_{elastic}\right)dV \quad \text{(S3)}$$

In Eq. (4), the Landau free energy density f_{bulk} is given by

$$\begin{aligned} f_{bulk} = {} & \alpha_1\left(P_1^2+P_2^2+P_3^2\right)+\alpha_{11}\left(P_1^4+P_2^4+P_3^4\right)+\alpha_{12}\left(P_1^2P_2^2+P_1^2P_3^2+P_2^2P_3^2\right) \\ & +\alpha_{111}\left(P_1^6+P_2^6+P_3^6\right)+\alpha_{112}\left(P_1^4P_2^2+P_1^4P_3^2+P_2^4P_3^2+P_2^4P_1^2+P_2^2P_3^4+P_1^2P_3^4\right)+\alpha_{123}P_1^2P_2^2P_3^2 \\ & +\alpha_{1111}\left(P_1^8+P_2^8+P_3^8\right)+\alpha_{1112}\left(P_1^6P_2^2+P_1^6P_3^2+P_2^6P_3^2+P_2^6P_1^2+P_1^2P_2^6+P_1^2P_3^6\right) \\ & +\alpha_{1122}\left(P_1^4P_2^4+P_1^4P_3^4+P_2^4P_3^4\right)+\alpha_{1123}\left(P_1^4P_2^2P_3^2+P_1^2P_2^4P_3^2+P_1^2P_2^2P_3^4\right) \end{aligned} \quad \text{(S4)}$$

where a_i, a_{ij}, a_{ijk}, and a_{ijkl} are the Landau coefficients of the polarization. For NNO film, the values are adopted from the literature:[1] $a_1 = -6.44\times10^7$ J m C^{-2} , $a_{11} = 9\times10^7$ J m^5 C^{-4} ,

$a_{12} = 1.4\times10^{9}$ J m^{5} C^{-4} , $a_{111} = 3.28\times10^{9}$ J m^{9} C^{-6} , $a_{112} = -3.46\times10^{9}$ J m^{9} C^{-6} , $a_{123} = -3.51\times10^{9}$ J m^{9} C^{-6} , $a_{1111} = 3.07\times10^{10}$ J m^{13} C^{-8} , $a_{1112} = 1.48\times10^{9}$ J m^{13} C^{-8} , $a_{1122} = 4.2\times10^{10}$ J m^{13} C^{-8}, and $a_{1123} = 6.2\times10^{10}$ J m^{13} C^{-8}; for the $SrTiO_3$, the values are from the literature:[2] $a_1 = 2.6353[\coth(42/T) - 0.90476]\times10^{7}$, $a_{11} = 1.696\times10^{9}$, $a_{12} = 1.373\times10^{9}$, $c_{11} = 3.36\times10^{11}$, $c_{12} = 1.07\times10^{11}$, $c_{44} = 1.27\times10^{11}$, $Q_{11} = 0.066$, $Q_{12} = -0.0135$, $Q_{44} = 0.0096$.

The gradient energy density is expressed as

$$\begin{aligned} f_{\text{gradient}} = {} & \frac{1}{2}G_{11}\left(P_{1,1}^2 + P_{2,2}^2 + P_{3,3}^2\right) + G_{12}\left(P_{1,1}P_{2,2} + P_{1,1}P_{3,3} + P_{2,2}P_{3,3}\right) \\ & + \frac{1}{2}G_{44}\left[\left(P_{1,2} + P_{2,1}\right)^2 + \left(P_{2,3} + P_{3,2}\right)^2 + \left(P_{1,3} + P_{3,1}\right)^2\right] \\ & + \frac{1}{2}G_{44}^{'}\left[\left(P_{1,2} - P_{2,1}\right)^2 + \left(P_{2,3} - P_{3,2}\right)^2 + \left(P_{1,3} - P_{3,1}\right)^2\right] \end{aligned} \tag{S5}$$

where G_{11}, G_{12}, G_{44} and $G_{44}^{'}$ are the gradient energy coefficients. The electrostatic energy is calculated as

$$f_{\text{electrostatic}} = -P_i E_i + \frac{1}{2}\kappa_b \kappa_0 E_i E_i \, , \tag{S6}$$

where κ_b is the background dielectric constant of the material and E_i is the electric field.

The elastic energy density is given by

$$f_{\text{elastic}} = \frac{1}{2}c_{ijkl}\left(\varepsilon_{ij} - \varepsilon_{ij}^0 + \varepsilon_{ij}^{\text{misfit}}\right)\left(\varepsilon_{kl} - \varepsilon_{kl}^0 + \varepsilon_{kl}^{\text{misfit}}\right) \tag{S7}$$

where ε_{ij} is the total strain field and c_{ijkl} is the elastic stiffness tensor, c_{11} = 230 GPa, c_{12} = 90 GPa, c_{44} = 76 GPa for NNO, whereas c_{11} = 336 GPa, c_{12} = 107 GPa, c_{44} = 127 GPa for NNO. ε_{ij}^0 are the spontaneous strain related to the local polarization, given by

$$\begin{aligned} \varepsilon_{11}^0 &= Q_{11}P_1^2 + Q_{12}\left(P_2^2 + P_3^2\right) \\ \varepsilon_{22}^0 &= Q_{11}P_2^2 + Q_{12}\left(P_1^2 + P_3^2\right) \\ \varepsilon_{33}^0 &= Q_{11}P_3^2 + Q_{12}\left(P_1^2 + P_2^2\right) \\ \varepsilon_{23}^0 &= Q_{44}P_2P_3 \\ \varepsilon_{13}^0 &= Q_{44}P_1P_3 \\ \varepsilon_{12}^0 &= Q_{44}P_1P_2 \end{aligned} \tag{S8}$$

where Q_{ij} are the electrostrictive coefficients, taken as Q_{11} = 0.046 m^4 C^{-2}, Q_{12} = −0.058 m^4 C^{-2}, and Q_{44} = 0.032 m^4 C^{-2} for NNO and the nanopillar region, whereas Q_{11} = 0.066 m^4 C^{-2}, Q_{12} = −0.0135 m^4 C^{-2}, and Q_{44} = 0.0096 m^4 C^{-2} for the $SrTiO_3$ substrate. The strain field ε_{ij} is obtained by solving the elastic equilibrium equation,

$$\frac{\partial}{\partial x_j}\left(\frac{\partial f}{\partial \varepsilon_{ij}}\right)=0 \tag{S9}$$

where $\sigma_{ij}={\partial f}/{\partial \varepsilon_{ij}}$ is the stress field given by $\sigma_{ij}=c_{ijkl}\left(\varepsilon_{kl}-\varepsilon_{kl}^{0}\right)$.

The model size of the simulated system is taken as $L_a \times L_b \times L_c$ = 82 × 82 × 78 nm^3, which is discretized into a three-dimensional mesh of 50 × 50 × 48 grids. The 2 layers of grids along the thickness direction of the film consist of a $SrTiO_3$ substrate, with a middle film region of 20×20×78 nm^3 set to be nanopillar region. Periodic boundary conditions are employed along the in-plane directions of the film for solving the polarization, and the electric field. A short-circuit boundary condition is employed at the top and bottom surfaces of the film with a fixed electric potential for solving the Maxwell equation,

$$\frac{\partial}{\partial x_i}\left(\frac{\partial f}{\partial E_i}\right)=\sigma \tag{S10}$$

where σ is the free charge in the ferroelectric material.

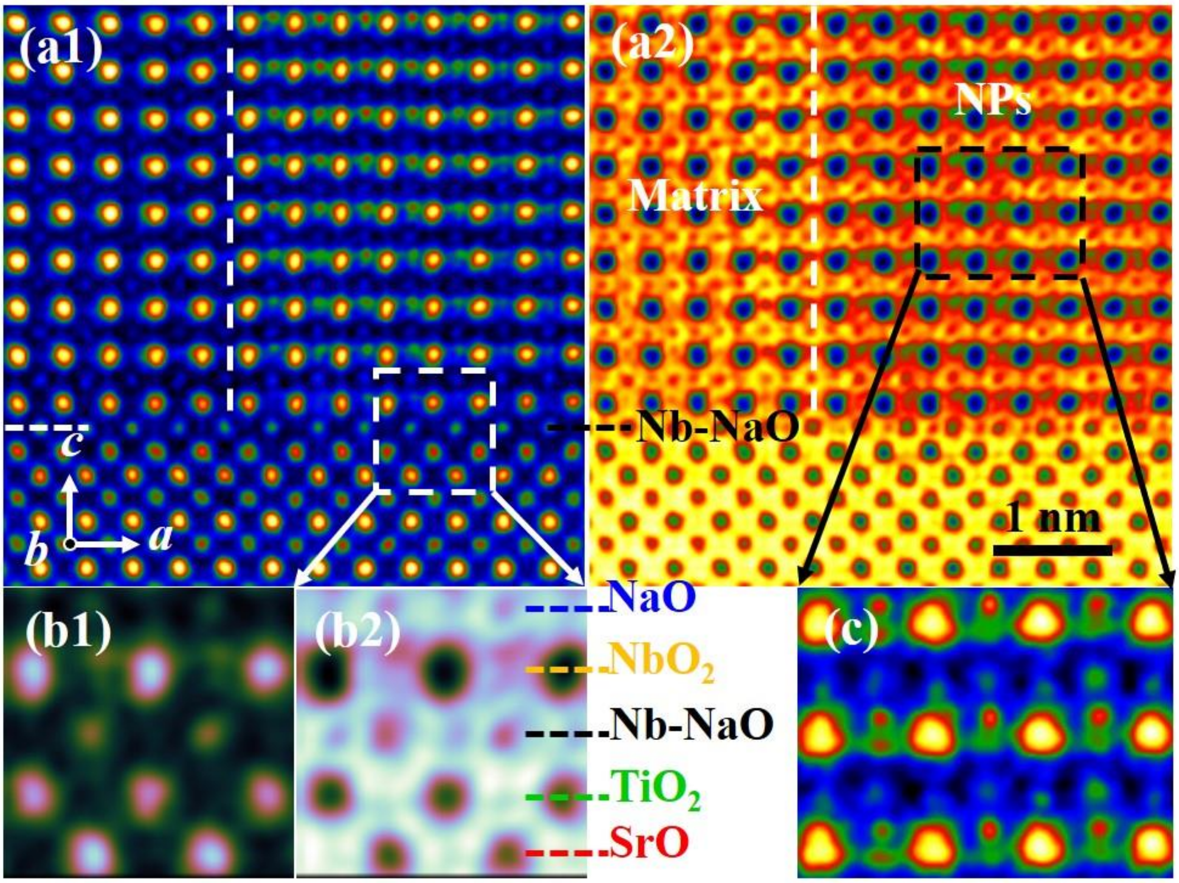

Figure S1. (a1, a2) Atomically resolved STEM HAADF and ABF images showing one antiphase-like boundary and the NNO/ STO interface. (b1, b2) Enlarged images focusing on the interface. (c) Enlarged image from (a2) focusing on the nanopillar.

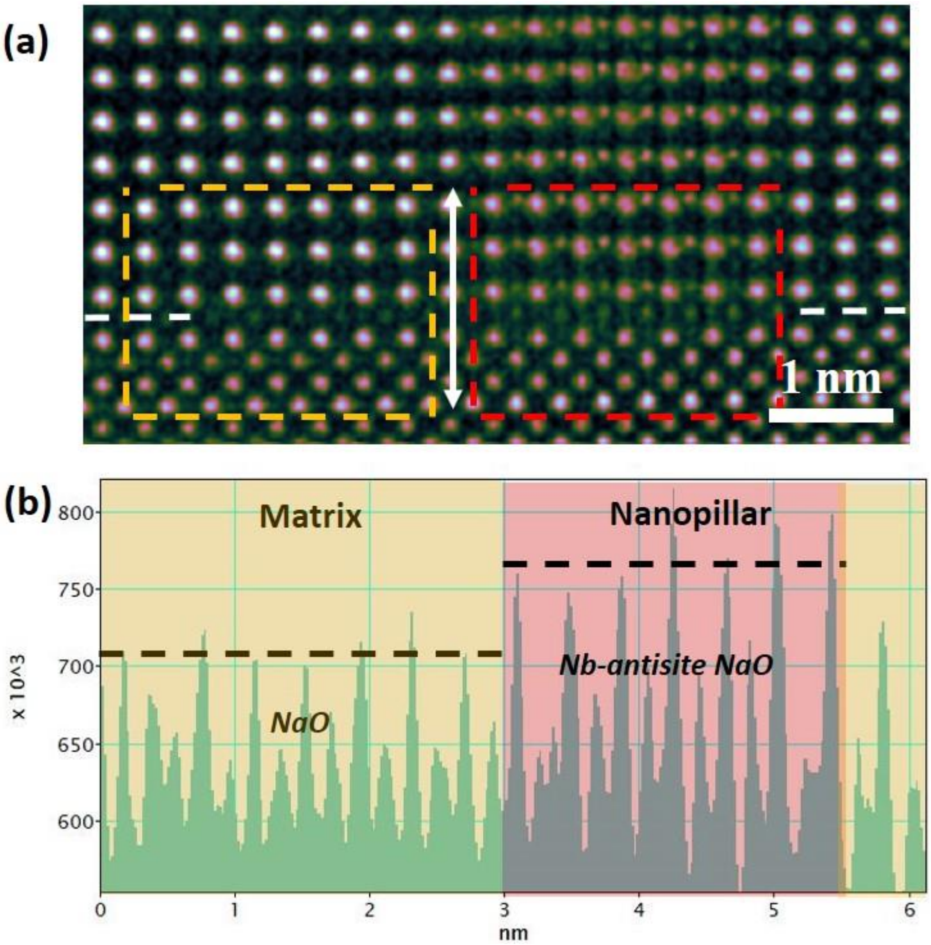

Figure S2. (a) Atomically resolved STEM HAADF image showing OOP boundaries and the NNO/STO interface. (b) Intensity profile from the while dashed line shown in (a).

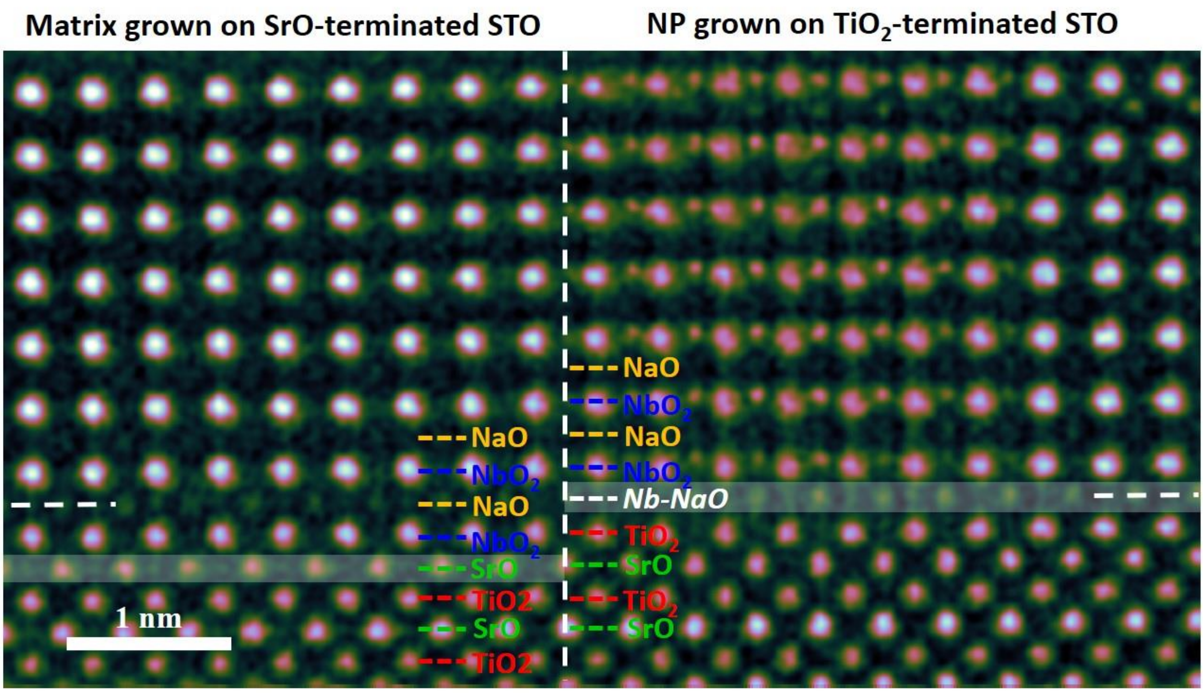

Figure S3. Atomically resolved STEM HAADF image showing the two types of interfaces, the left part is matrix grown on SrO-terminated STO, while the right part is a polar nanopillar grown on TiO_2-terminated STO. In the left region, the interface stacking sequence is TiO_2-SrO-NbO_2 (mixed with Sr or Ti since it is less bright than the next NbO_2 layer)-NaO-NbO2. In the right region, the stacking sequence is TiO_2-SrO-TiO_2-NaO (mixed with Nb, Sr, or Ti since it is brighter than the next NaO layer)-NbO_2-NaO.

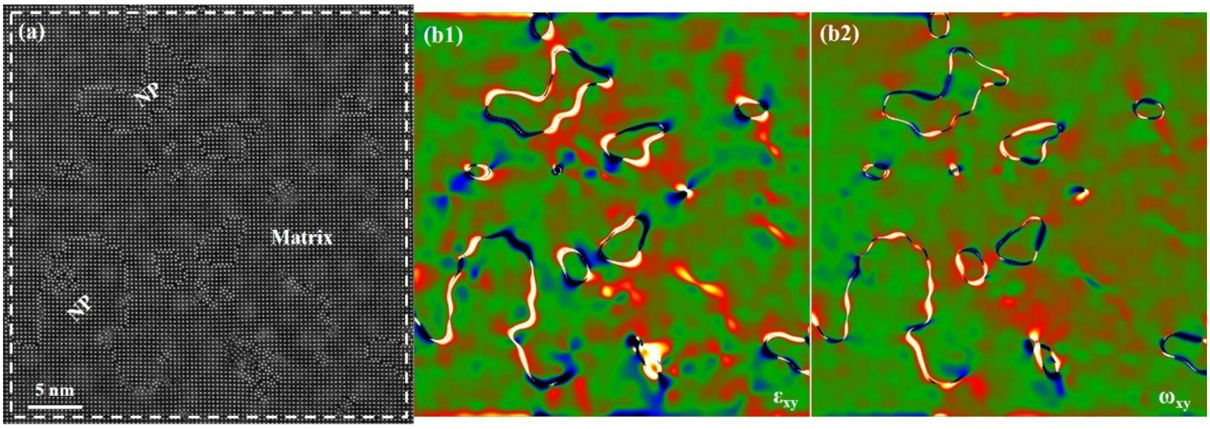

Figure S4. (a) Plan-view STEM HAADF image of NP-NNO/Nb-STO showing a high density of polar nanopillars. (b1, b2) Strain analysis of (a), ε_{xy} is strain tensor and ω_{xy} is rotation (in radians and anti-clockwise positive).

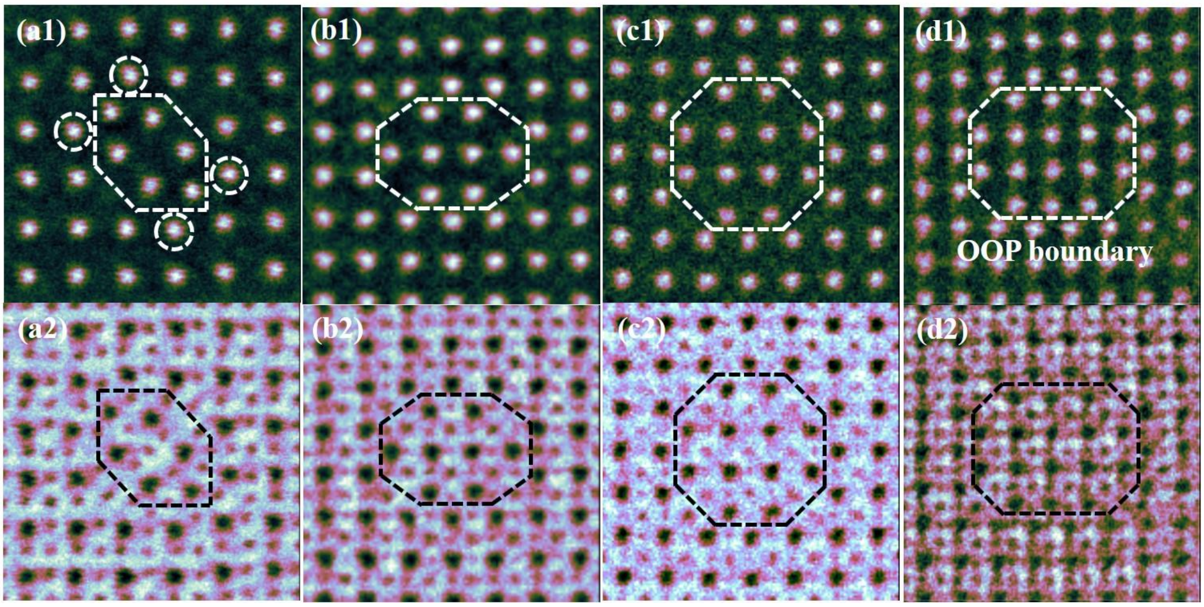

Figure S5 (a1, a2, b1, b2, c1, c2, d1 d2). Enlarged STEM HAADF/ABF images of one nanopillar with 6 (**a1, a2)**, 8 **(b1, b2)**, 12 **(c1, c2)**, 16 (**d1 d2)** Nb atoms, respectively.

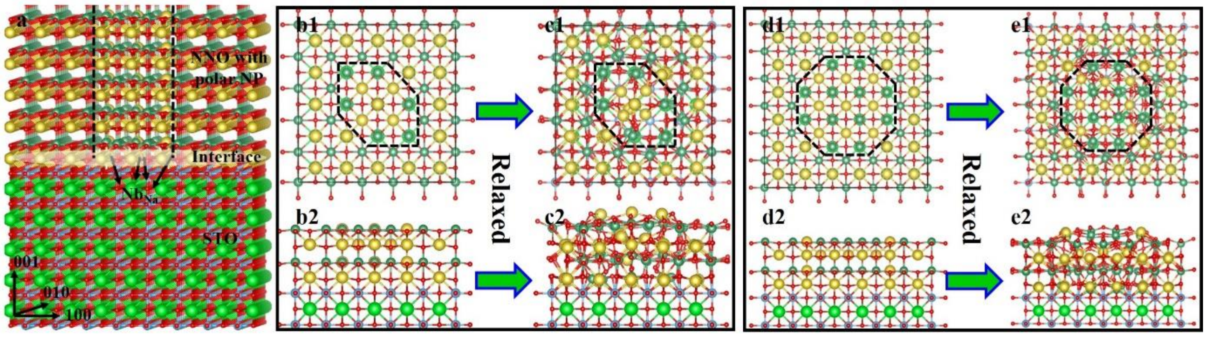

Figure S6. (a) The 3D structural model with one polar nanopillar and the NNO/Nb-STO interface. (b1, b2), Initial structural model of one polar nanopillar with 6 Nb atoms and the NNO/STO interface, viewed along [001] and [100] axes. (c1, c2) Relaxed structural models of (a1, a2). (d1, d2) Initial structural model of one polar nanopillar with 12 Nb atoms and the NNO/Nb-STO interface, viewed along [001] (plan view) and [100] (cross-section view) axes. (e1, e2) Relaxed structural models of (d1, d2).

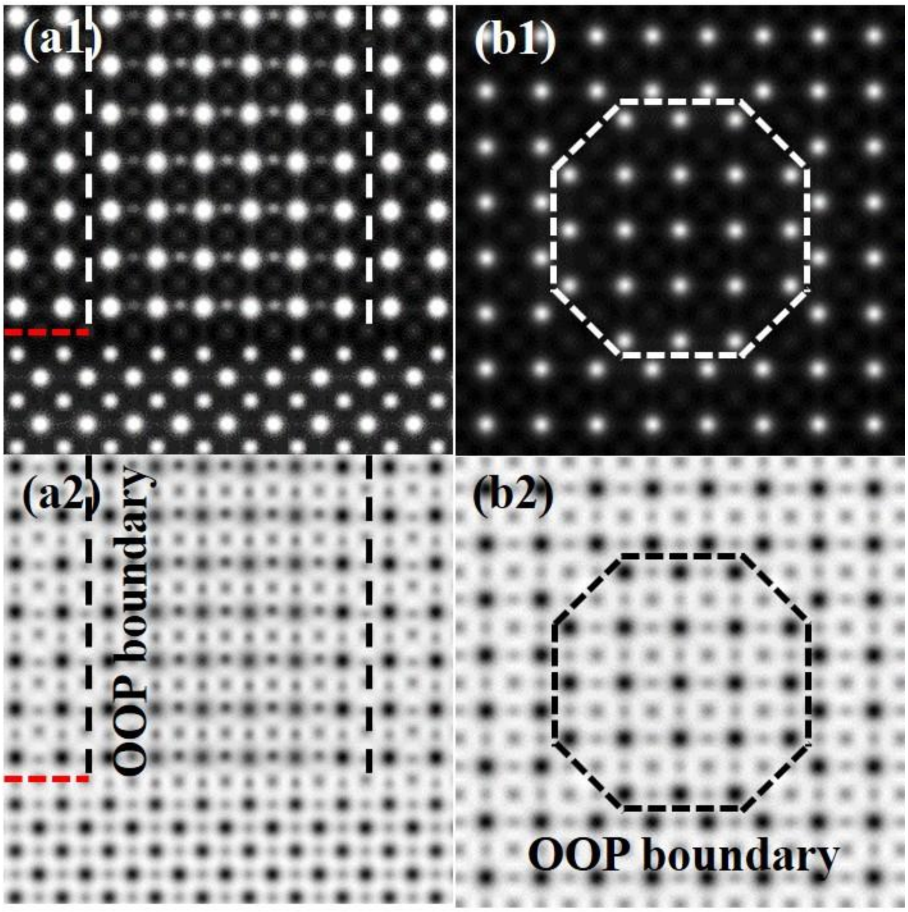

Figure S7. Simulated STEM HAADF/ABF images of NP-NNO/STO with one nanopillar.

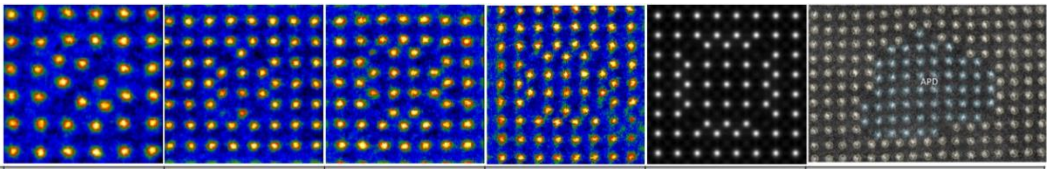

Antisite phase	6 Nb atoms	8 Nb atoms	12 Nb atoms	16 Nb atoms	21 Nb atoms	56-atom
Nb numbers in nanopillar	6	8	12	16	21	56
Nb numbers in respective matrix	4	6	9	12	16	48
Nb effective valency	3.33+	3.75+	3.75+	3.75+	3.71+	4.29+

Figure S8. Enlarged images showing four antisite phases with 6, 8,12, and 56 Nb atoms. In comparison, the Nb atom numbers in the respective matrixes are 4, 6, 9, and 48, respectively. Then the effective Nb valency can be estimated as 3.33+, 3.75+, 3.75+, and 4.29+, respectively.

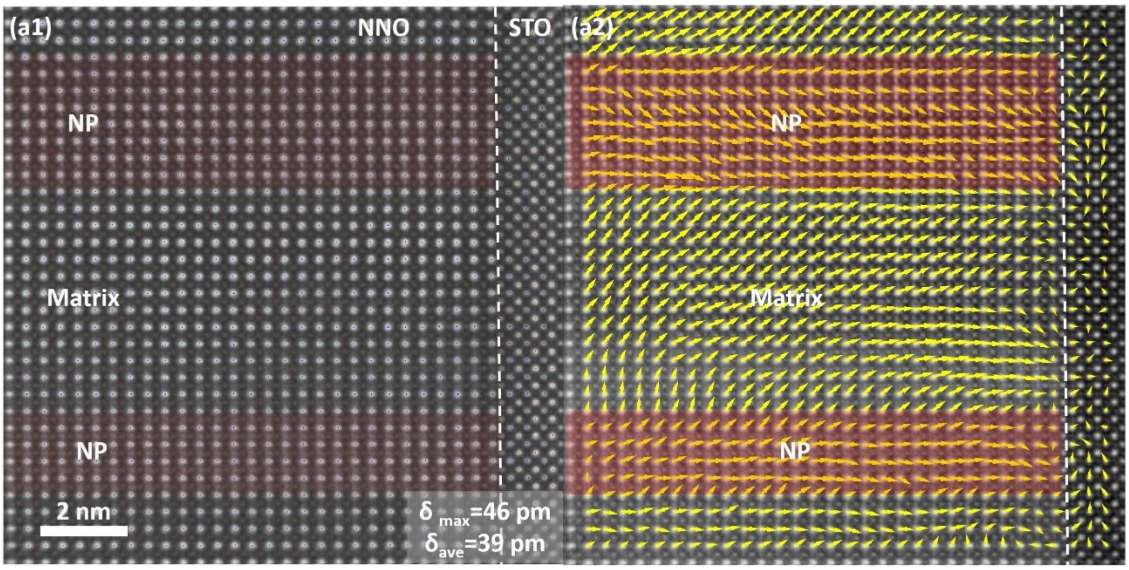

Figure S9. (a) Cross-section STEM HAADF image of NP-NNO/STO film, with peaks found. (b) Polar atom (Nb) displacement (i.e., polarization) arrow map overlaid on a the atom displacements calculated based on the center polar Nb atom displacement with respect to the corner Na atoms. The nanopillars have been coloured red.

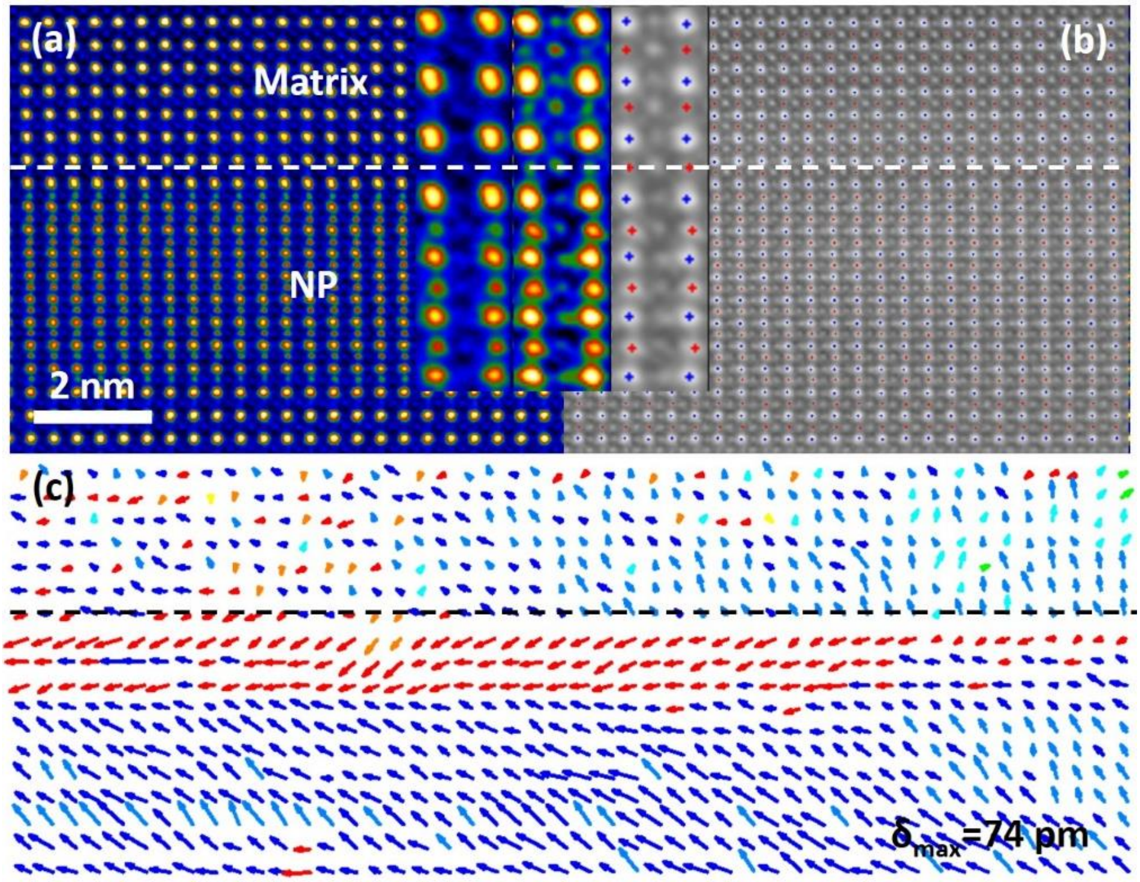

Figure S10. (a) A STEM HAADF image and (b) the respective STEM ABF (contrast reversed) image, with enlargements showing the antiphase-like boundary between the polar nanopillar and the matrix. (c) Colorized arrow map, the atom displacement calculated based on the nanopillar-Nb atoms in the nanopillar region or the matrix-O atoms in the matrix with respect to their nearest matrix Nb atoms, as marked in (b).

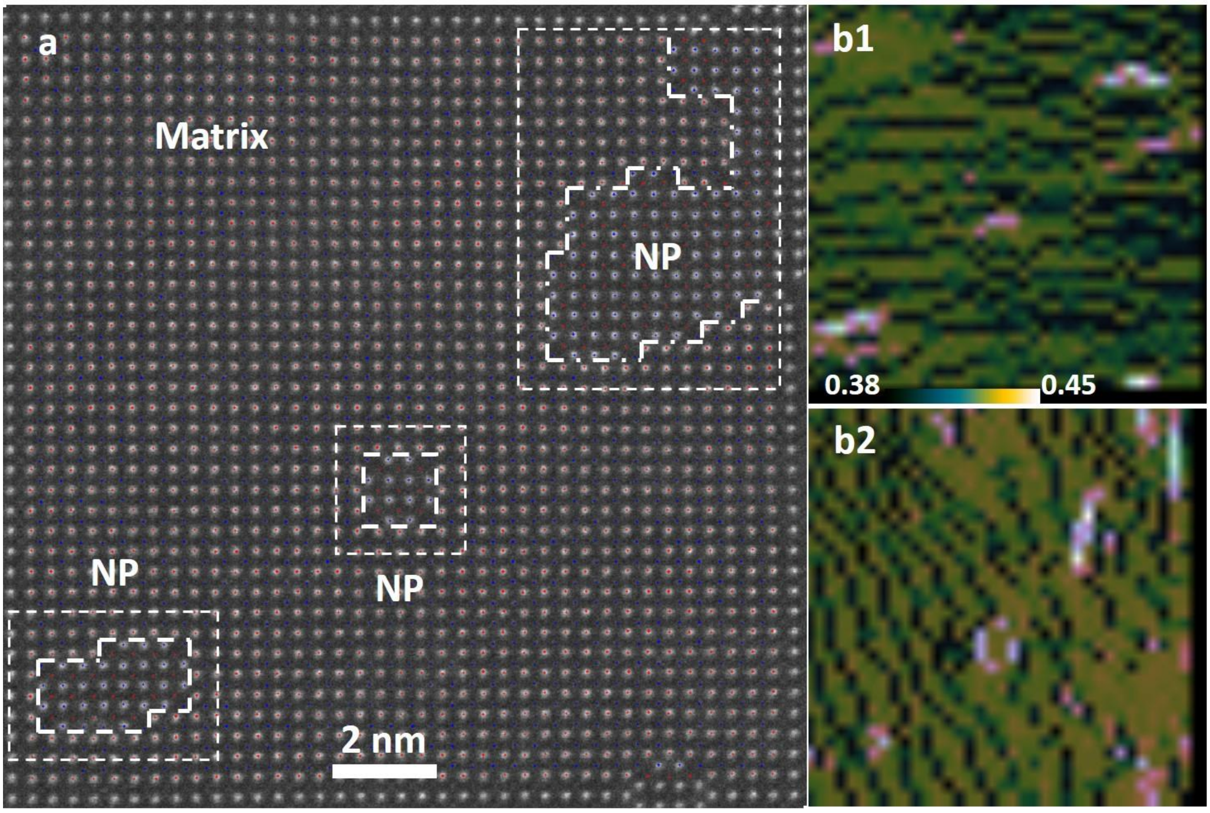

Figure S11. Strain analysis. a Plan-view STEM HAADF image of NP-NNO film, where three nanopillars are marked. (**b1**, **b2**), Lattice parameter maps along the X and Y directions, the unit of the scale is nm.

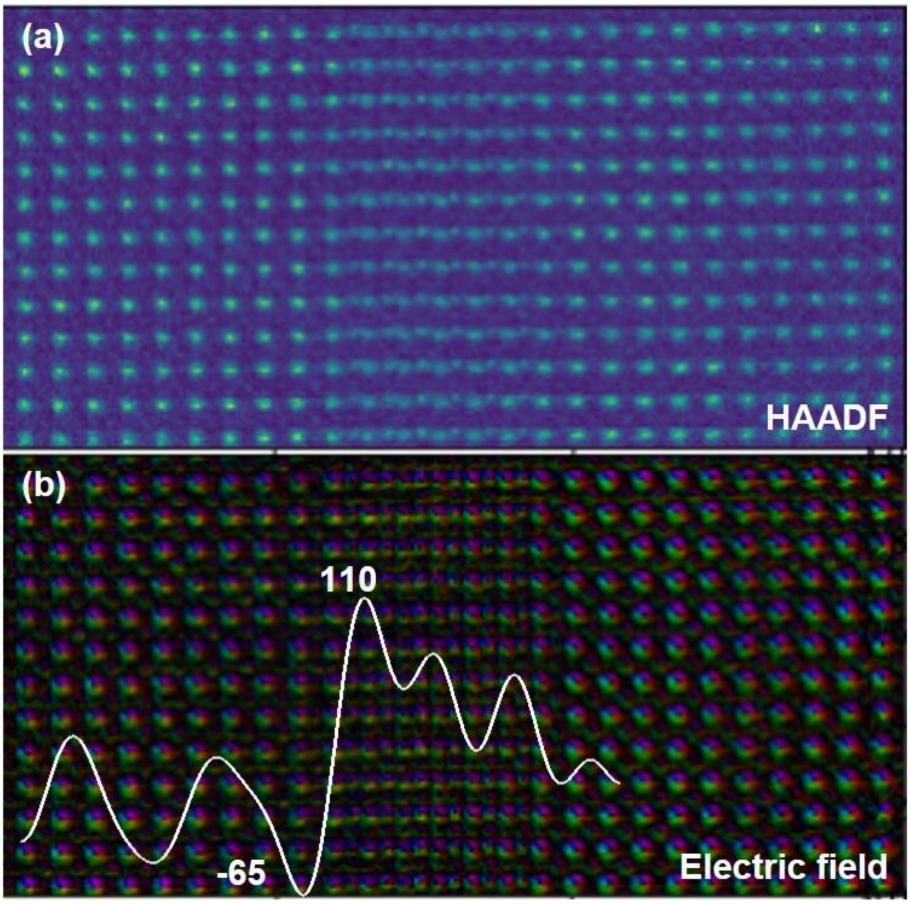

Figure S12. STEM-DPC for the charged OOP boundary. (a) STEM HAADF image showing one nanopillar; **(b)** the corresponding generated distribution of the electric fields, with averaged line profile of the electric field along the horizontal direction. (c) The computed charge density according to the electric fields.

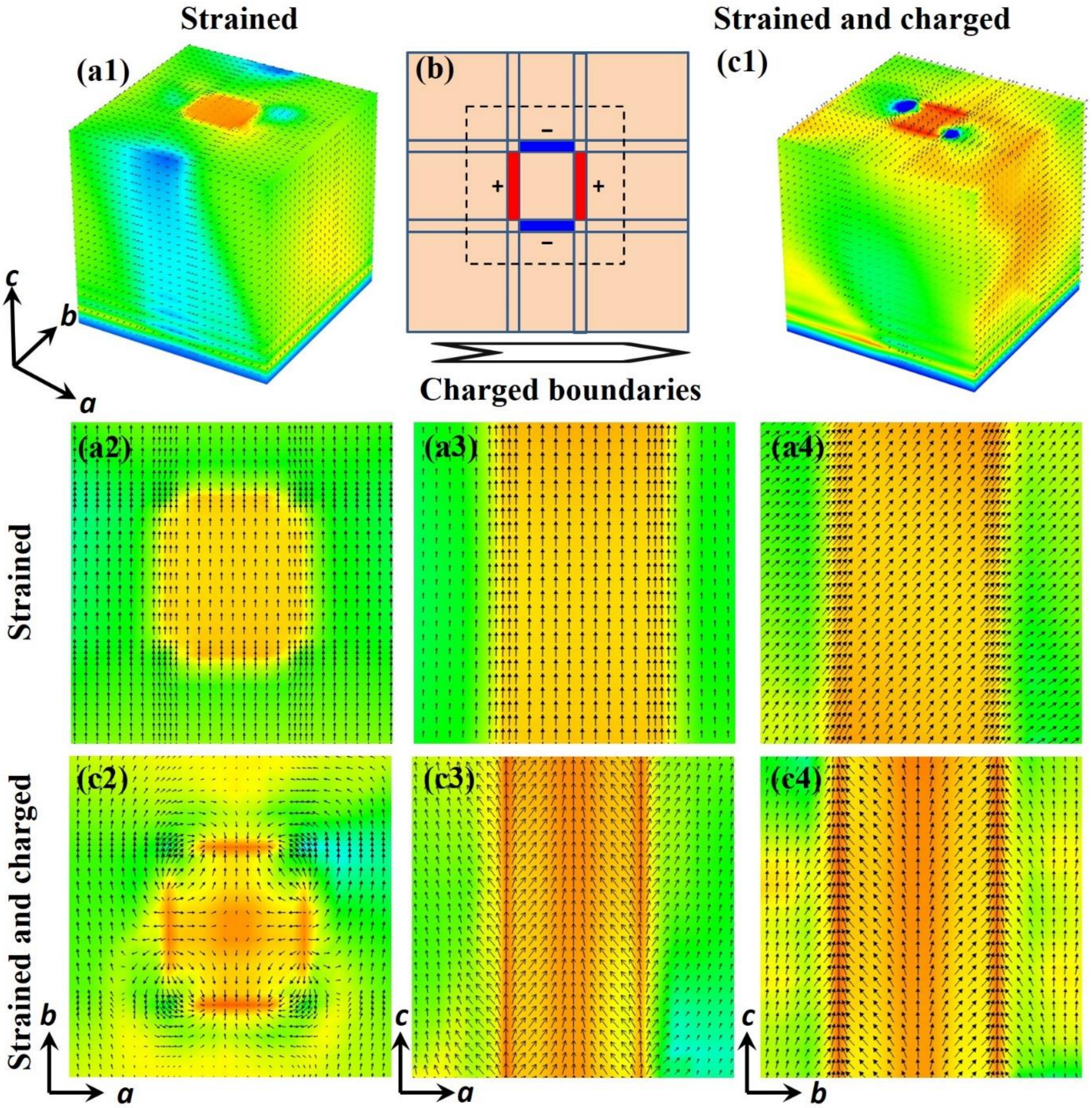

Figure S13. Phase-field simulation of strained and charged antiphase-like boundaries. (a) 3D view of the domain structure in a NNO film with a strained nanopillar. (a2-a4) Images of 2D slices along *c*, *b*, *a* axes respectively at the middle section of (a1). (b) Schematic showing the charges at the antiphase-like boundaries. (c1) 3D view of domain structure in a NNO film with a strained and charged nanopillar. (c2-c4) 2D slice images along *c*, *b*, *a* axes respectively at the middle section of (c1). Regions with different colors indicate ferroelectric domains with different polarization including orientation and magnitude.

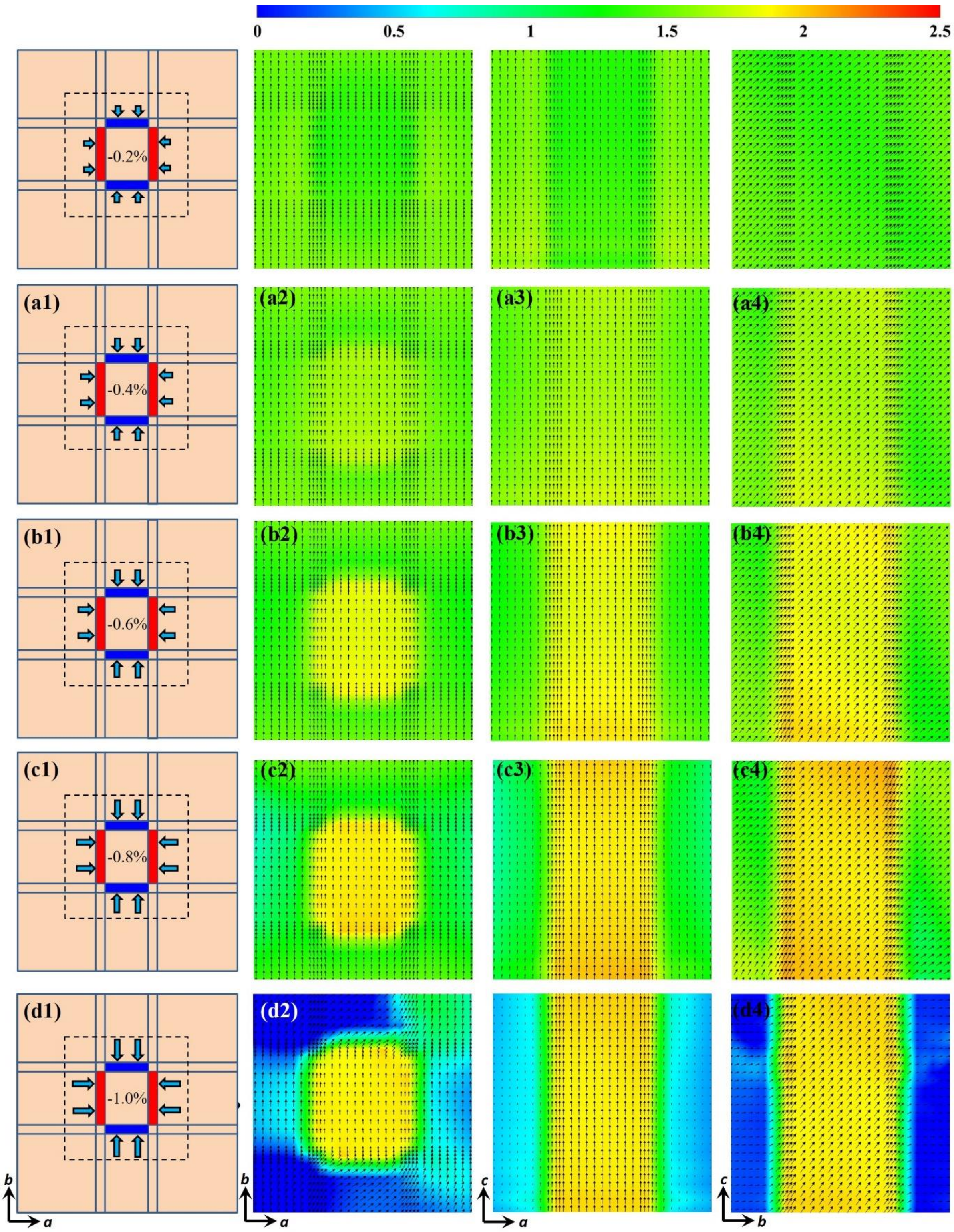

Figure S14. Phase field simulation of different misfit strain in the nanopillar and anti-site boundary region. (a1)-(a4) misfit strain -0.4%, (b1)-(b4) misfit strain -0.6%, (c1)-(c4) misfit strain -0.8%, (d1)-(d4) misfit strain -1.0%. There is no charge setting for all cases.

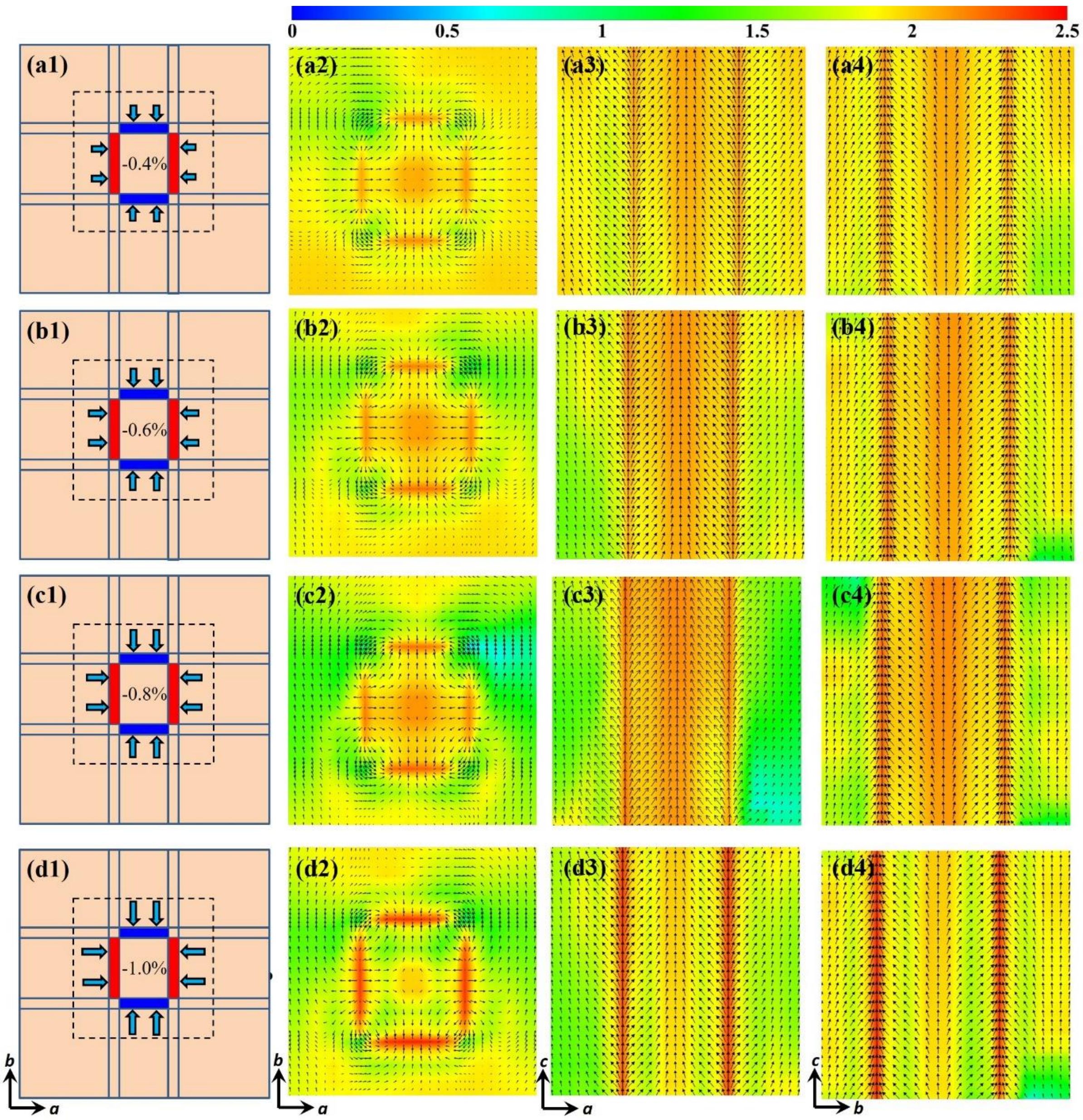

Figure S15. Phase field simulation of different misfit strain in the nanopillar and antiphase-like boundary region. (a1)-(a4) misfit strain -0.4%, (b1)-(b4) misfit strain -0.6%, (c1)-(c4) misfit strain -0.8%, (d1)-(d4) misfit strain -1.0%. The charge density of all cases is 2.0/-2.0.

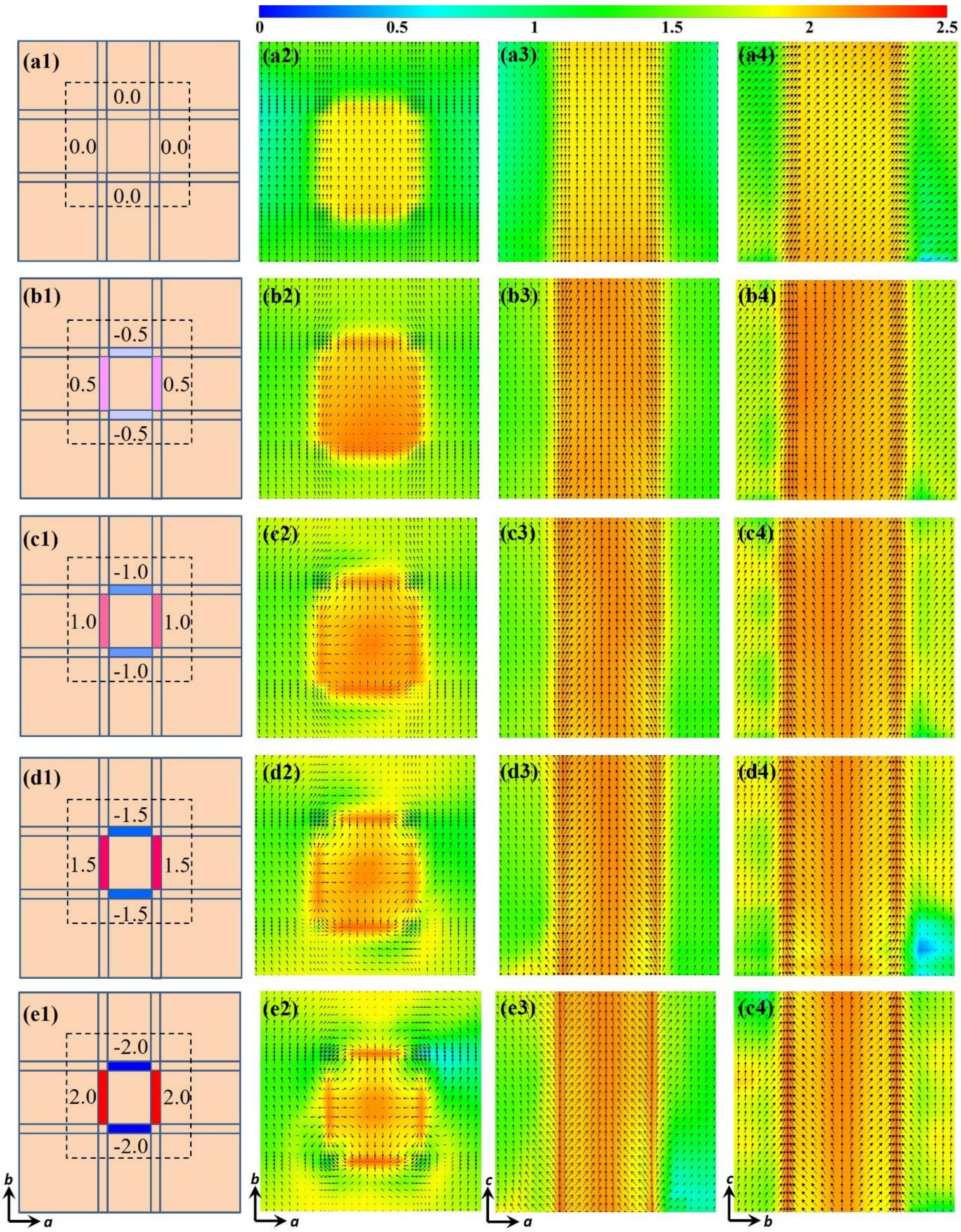

Figure S16. Phase field simulation of different charge density along the antiphase-like boundaries. (a1)-(a4) charge density 0.0, (b1)-(b4) charge density 0.5/-0.5, (c1)-(c4) charge density 1.0/-1.0, (d1)-(d4) charge density 1.5/-1.5, (e1)-(e4) charge density 2.0/-2.0.

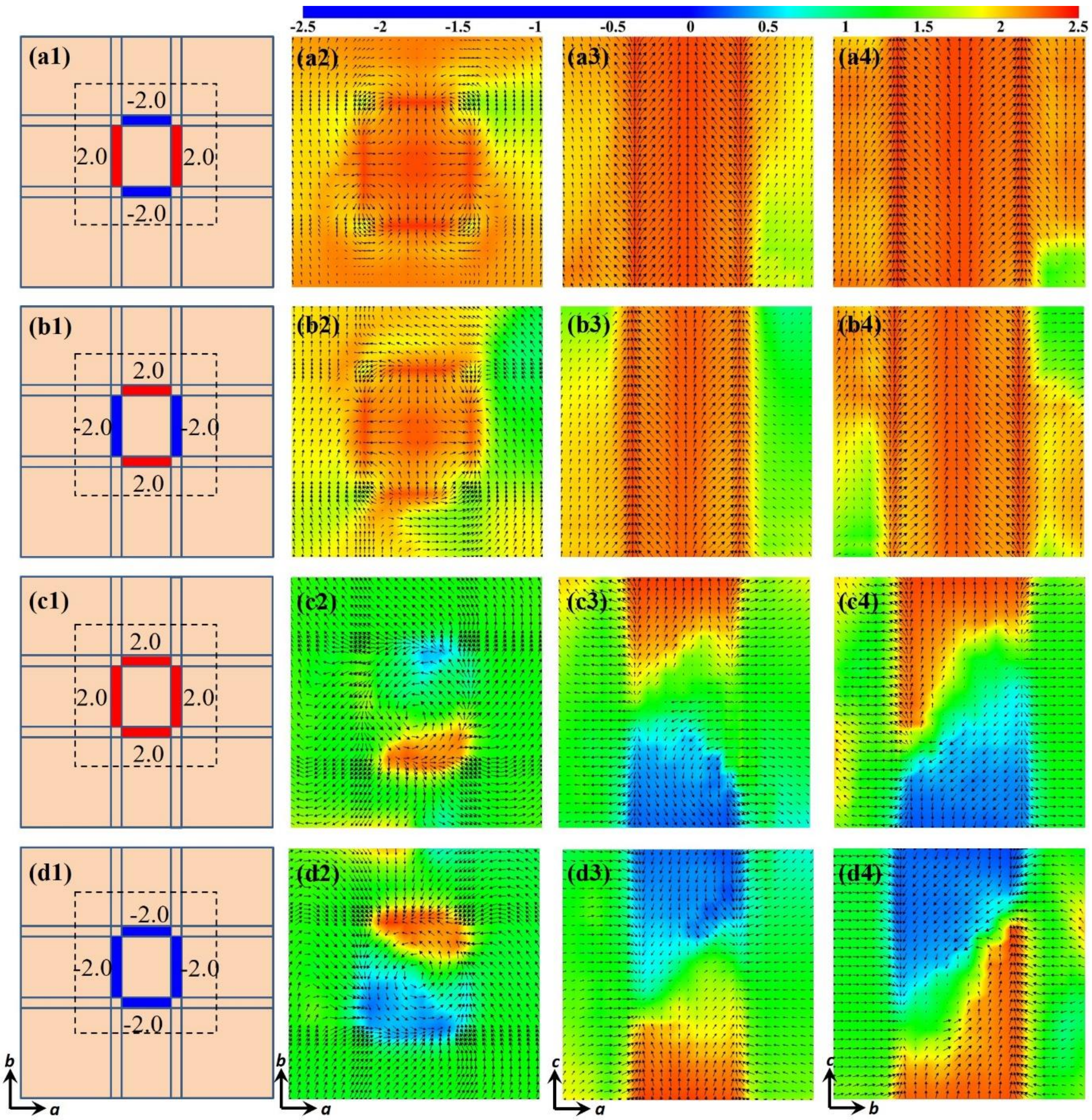

Figure S17. Phase-field simulation of different charge distributions along the antiphase-like boundaries.

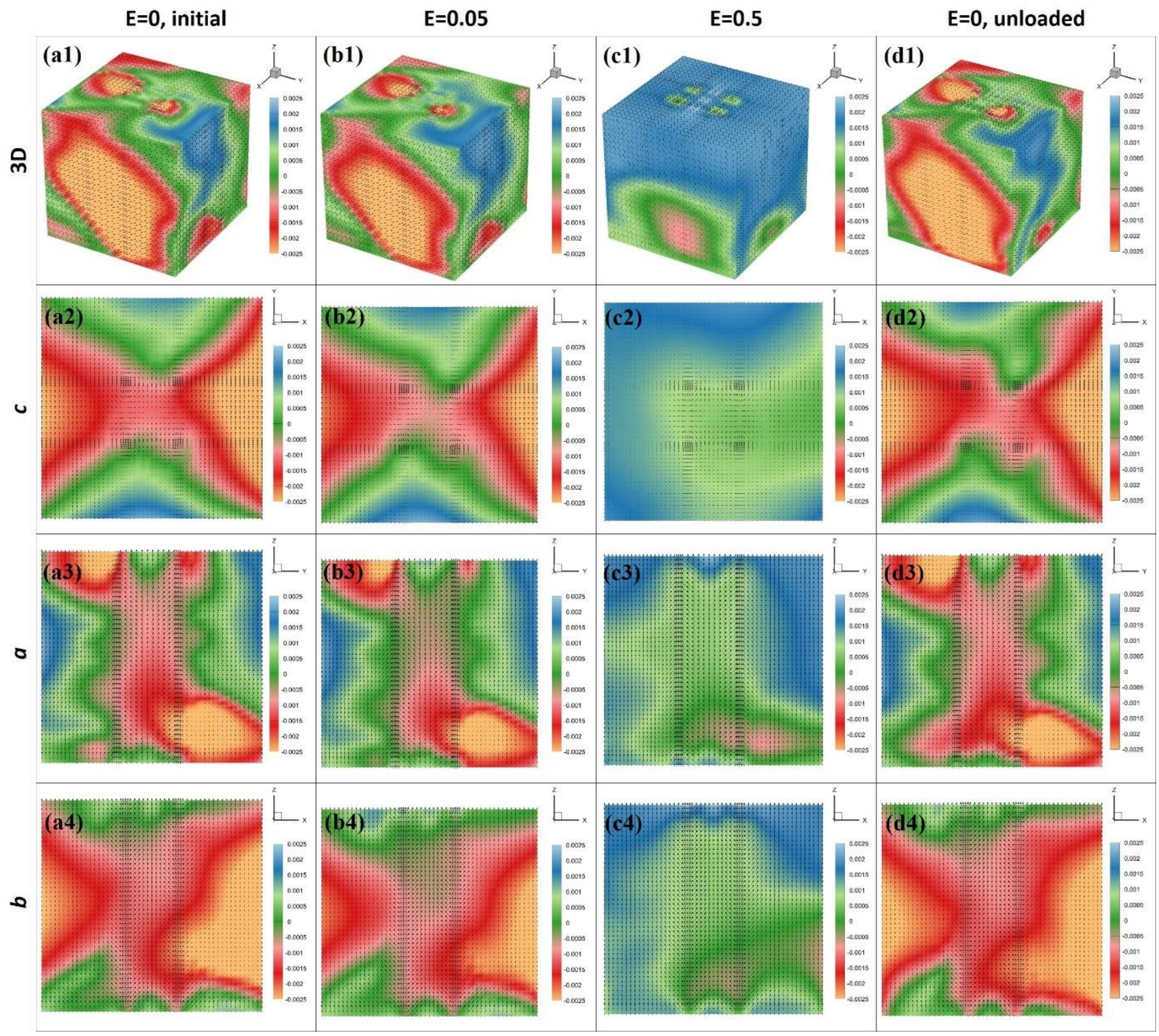

Figure S18. Phase-field simulation of strain map under an electric field. (a1-a4), (b1-b4), (c1,-c4) and (d1-d4), 3D and 2D strain maps of the strained and charged NPR-NNO film at the initial state and under an applied electric field of 0.05, 0.5 a. u. (arbitrary unit) respectively, as well as the unloaded state (back to the initial state).

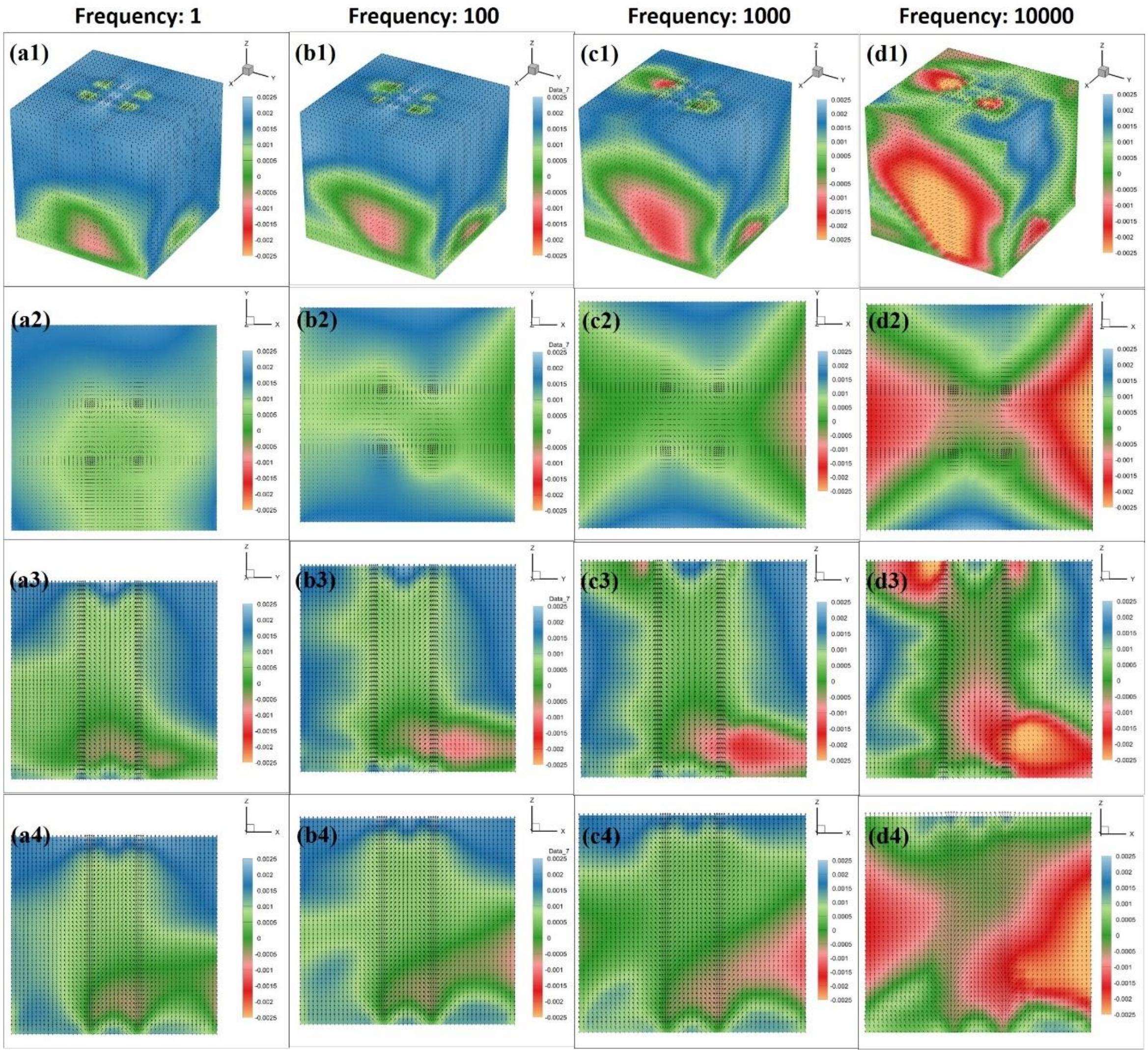

Figure S19. Phase-field simulation of strain map under A.C. electric field (0.05 a.u.) with different frequencies. (a1-a4), (b1-b4), (c1,-c4) and (d1-d4), 3D and 2D strain maps at frequency of 1, 100, 1000, 10000 a. u. (arbitrary unit) respectively.

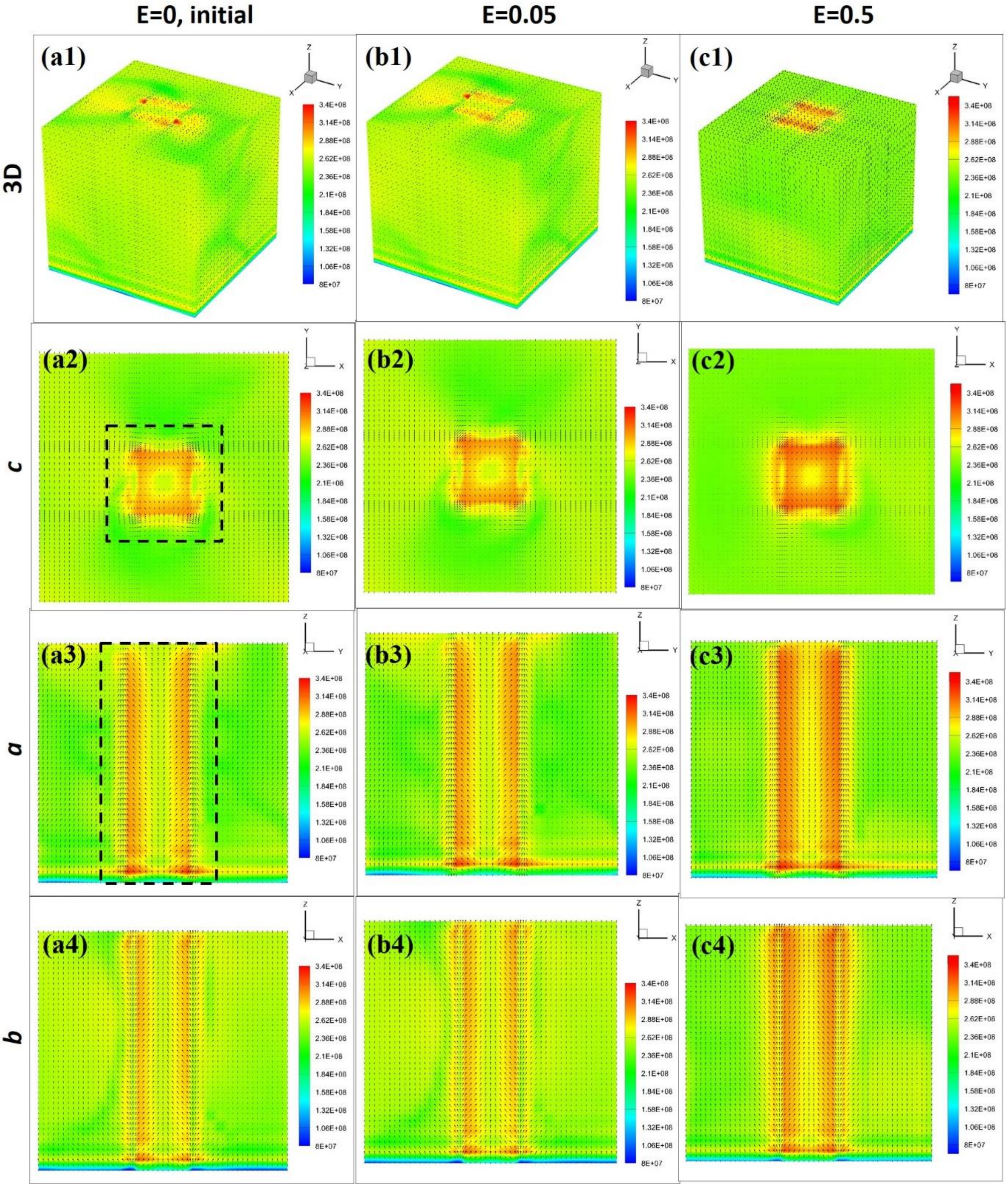

Figure S20. Phase-field simulation of depolarization field at E=0, 0.05, and 0.5 a.u.

Reference

1 Liu, H. *et al.* Giant piezoelectricity in oxide thin films with nanopillar structure. *Science* **369**, 292-297, doi:10.1126/science.abb3209 (2020).

2 Li, Y. L. *et al.* Prediction of ferroelectricity in $BaTiO_3$/$SrTiO_3$ superlattices with domains. *Applied Physics Letters* **91**, 112914, doi:10.1063/1.2785121 (2007).

3 Wu, H.-H. *et al*. Pseudo-first-order phase transition for ultrahigh positive/negative electrocaloric effects in perovskite ferroelectrics. *Nano Energy* **16**, 419-427, doi:10.1016/j.nanoen.2015.06.030 (2015).

4 Wang, J. et al.. Phase-field simulations of ferroelectric/ferroelastic polarization switching. *Acta Materialia*, **52**, 749-764, doi:10.1016/j.actamat.2003.10.011 (2004).

5 Pohlmann, H. *et al*. A thermodynamic potential and the temperature-composition phase diagram for single-crystalline $K_{1-x}Na_xNbO_3$ ($0\leq x\leq 0.5$). *Applied Physics Letters* **110**, 102906, doi:10.1063/1.4978360 (2017).

6 Song, D. Electronic and plasmonic phenomena at nonstoichiometric grain boundaries in metallic $SrNbO_3$. *Nanoscale*, **12**, 6844-6851, doi:10.1039/C9NR10221C (2020)

7 Huang, H. Phase stability and fast ion transport in P2-type layered $Na_2X_2TeO_6$ (X = Mg, Zn) solid electrolytes for sodium batteries. *Journal of Materials Chemistry A*, **8**, 22816-22827, doi:10.1039/D0TA07469A (2020)
